# Handbook of Zoonoses: Identification and Prevention

**DOI:** 10.3201/eid1402.071283

**Published:** 2008-02

**Authors:** M. Kathleen Glynn

**Affiliations:** * Centers for Disease Control and Prevention, Atlanta, Georgia, USA

## Abstract

Handbook of Zoonoses: Identification and Prevention

In “Handbook of Zoonoses: Identification and Prevention,” authors Joann Colville and David L. Berryhill laud their book as an ideal reference for veterinarians, veterinary technicians, and professional students, and as a general resource for healthcare professionals to help them understand and manage zoonotic diseases. Information on common, and currently topical, zoonoses are included; the book addresses diseases caused by bacteria, viruses, parasites, fungi, and prions. For each disease, a common set of concepts are covered: the degree of illness and death associated with the disease; the etiology, hosts, and routes of transmission; a brief description of the disease manifestation in various animal species and in humans; general guidance for treatment in both animals and humans; and recommendations for prevention. Although the authors present some information on diseases that occur outside the United States, this handbook focuses more on diseases in the United States that readers may come into contact with or hear about on the news.

The terminology used to cover zoonotic diseases may be useful to healthcare professionals because basic information is provided in easily understandable language for lay patients and clients. As an actual source of information for healthcare professionals, however, this handbook is not as useful as other available texts. The book covers aspects of many of these diseases somewhat superficially and makes generalizations that could be misleading when examined in greater depth. This weakness is exacerbated by the fact that no references are provided, either as source data for citation or as pointers for readers looking for more information on a particular disease. Given the lack of overall detail in the handbook, this is a significant shortcoming. And, while an overall goal of the book is to provide information to help healthcare professionals manage zoonotic diseases, the authors do not present specific treatment guidance or common differential diagnoses for the diseases covered. Instead, the authors provide the occasional piece of trivia about certain diseases covered in the handbook that a lay reader may find engaging—that Ted Nugent wrote a song called “Cat Scratch Fever” and that rumors exist of a “Hollywood Tapeworm Diet.” Overall, this handbook may be best suited for the lay person who has an interest in zoonotic diseases and some preexisting knowledge of disease transmission and pathogenesis.

